# Mental health surveillance of German adults in the COVID-19 pandemic: trends in depressive symptoms: Lena Walther

**DOI:** 10.1093/eurpub/ckac131.487

**Published:** 2022-10-25

**Authors:** L Walther, S Junker, C Kersjes, S Damerow, S Mueters, J Thom, E Mauz

**Affiliations:** Epidemiology and Health Monitoring, Robert Koch Institute, Berlin, Germany

## Abstract

**Background:**

While literature on mental health in the COVID-19 pandemic has grown rapidly, studies reporting on developments beyond the first wave using continuous, population-based data are still scarce. We examined monthly estimates of depressive symptom levels in Germany’s adult population covering almost two years of the pandemic and the year prior.

**Methods:**

We analyzed representative data from two population-based telephone surveys of German adults: “German Health Update (GEDA)” and “COVID-19 vaccination rate monitoring in Germany (COVIMO).” Core symptoms of depression measured using the Patient Health Questionnaire-2 (PHQ-2) were observed in approximately 1,000 randomly sampled participants monthly from April 2019 to December 2021. We estimated three-month moving means and proportions as well as smoothing curves to produce time series graphs. Statistical comparisons between specific time periods were used to verify results of visual inspection. Analyses were stratified by gender, age and level of education to assess potential time trend differences between subgroups.

**Results:**

Both the mean population depressive symptom score and the proportion of the population with a positive PHQ-2 depression screen first decreased to below 2019 levels between the first wave and summer of the pandemic and then increased from autumn 2020, reaching levels significantly above 2019 in 2021 and remaining elevated. 2021 saw a 2.2% increase in positive screens compared to 2019. Women, the youngest and eldest adults, and those with a high level of education experienced a particular increase in depressive symptoms between 2019 and 2021. However, we found no corresponding changes in symptom level differences between population subgroups.

**Conclusions:**

Our finding of elevated depressive symptoms among Germany’s adults following an increase in the second wave of the pandemic demonstrate the importance of continued surveillance to assess the further development of mental health in the ongoing crisis.

**Key messages:**

• Monthly data from April 2019 to December 2021 suggests that depressive symptoms decreased at the start of the pandemic and then increased from autumn 2020, reaching levels above 2019 in 2021.

• Continued mental health surveillance is needed to assess the further development of mental health indicators in the ongoing crisis and its aftermath.

